# Cryoablation and Immunotherapy: An Enthralling Synergy to Confront the Tumors

**DOI:** 10.3389/fimmu.2019.02283

**Published:** 2019-09-24

**Authors:** Chakradhar Yakkala, Cheryl Lai-Lai Chiang, Lana Kandalaft, Alban Denys, Rafael Duran

**Affiliations:** ^1^Department of Diagnostic and Interventional Radiology, Lausanne University Hospital, Lausanne, Switzerland; ^2^Vaccine Development Laboratory, Ludwig Center for Cancer Research, University of Lausanne, Lausanne, Switzerland; ^3^Department of Oncology, Ludwig Institute for Cancer Research, University of Lausanne, Lausanne, Switzerland

**Keywords:** cryo, ablation, immunotherapy, tumors, freezing, treatment

## Abstract

Treatment of solid tumors by ablation techniques has gained momentum in the recent years due to their technical simplicity and reduced morbidity as juxtaposed to surgery. Cryoablation is one of such techniques, known for its uniqueness to destroy the tumors by freezing to lethal temperatures. Freezing the tumor locally and allowing it to remain *in situ* unleashes an array of tumor antigens to be exposed to the immune system, paving the way for the generation of anti-tumor immune responses. However, the immune responses triggered in most cases are insufficient to eradicate the tumors with systemic spread. Therefore, combination of cryoablation and immunotherapy is a new treatment strategy currently being evaluated for its efficacy, notably in patients with metastatic disease. This article examines the mechanistic fabric of cryoablation for the generation of an effective immune response against the tumors, and various possibilities of its combination with different immunotherapies that are capable of inducing exceptional therapeutic responses. The combinatorial treatment avenues discussed in this article if explored in sufficient profundity, could reach the pinnacle of future cancer medicine.

## Introduction

Percutaneous image-guided ablation techniques are playing an increasing role in the management of patients with solid cancers. The minimally invasive nature of percutaneous ablations together with their proven antitumor efficacy have gained a wide acceptance in the medical community. In fact, percutaneous ablations have demonstrated to be analogous to surgery in achieving total eradication of small tumors with the added advantage of decreased morbidity (1, 2). While surgical resection extirpates the tumor, ablative therapies yield tumor cell death *in situ*. This unique feature offers a therapeutic opportunity as it uncovers to the immune system previously shielded tumor antigens. Indeed, the immunomodulatory abilities of percutaneous ablation therapies could be used as *in vivo* vaccination tools, and combinatorial approaches with immunotherapy could be beneficial for patients with widespread disease.

Commonly employed ablation therapies in the clinical setting are radiofrequency ablation (RFA), microwave ablation, high-intensity focused ultrasound and cryoablation. All these treatments operate on the principle of hyperthermia with the exception of cryoablation, which is a hypothermic modality that induces tissue damage by a freeze-thaw process. Of all the ablation techniques, cryoablation demonstrated the highest potential to elicit post-ablative immunogenic response. Chapman et al., performed cryoablation of healthy hepatic tissue in Sprague-Dawley rats that resulted in the appearance of pulmonary lesions, characterized by the presence of lymphocytes, neutrophils and foamy macrophage clusters. In contrast, these lesions were not observed in the rats subjected to hepatic RFA. Activation of nuclear factor-κB (NF-κB) in lung and liver tissues was prominent in the animals subjected to cryoablation, but not RFA. Concomitantly, higher serum cytokine levels of tumor necrosis factor (TNF)-α and macrophage inflammatory protein-2 were observed post cryoablation as opposed to RFA (3). In another similar study, hepatic cryoablation induced an increase in white blood cell counts, higher serum levels of aspartate/alanine aminotransferases and interleukin (IL)-6 as compared to RFA or laser induced thermotherapy, despite similar volume of hepatic damage achieved in all the three techniques of ablation (4). In a subsequent clinical study on tumors treated with cryoablation or RFA or microwave ablation, patients treated with cryoablation exhibited significantly elevated plasma IL-6 levels as compared to patients that received RFA or microwave ablation (5). This could be explained by the fact that heat-based therapies can cause protein denaturation, reducing the availability of intact immunogenic antigens, which can be circumvented by cryoablation, as demonstrated by a recent study employing Fourier-transform infrared spectroscopy (6).

## Brief History of Cryoablation in Tumors

Cryoablation refers to the technique of ablating the tissue by freezing to lethal temperatures followed by thawing, causing extensive tissue destruction. This technique is widely used to treat benign and malignant primary tumors (7, 8). Although the use of cold temperatures to treat wounds dates back to 3000 B.C. (9), its application to treat the tumors was first attempted by James Arnott in the nineteenth century. He successfully attempted the usage of cold temperatures by salt and ice solutions for the generation of local numbness prior surgical operations, as a replacement for chloroform inhalation. He reported that the freezing temperatures not only acted as a local anesthetic, but also impaired cancer cell viability that translated into patient's extended survival, and suggested cryoablation as an attractive therapeutic option for treating local tumors (9, 10). In 1930s, William Pusey started using liquid CO_2_ under high pressure, which upon release and expansion at the atmospheric pressure resulted in cooling effect and the subsequent formation of ice crystals (10). This is famously known as the Joule-Thomson effect, the principle underlying all the modern techniques of cryoablation. Around the same time, Irvine and Turnacliffe utilized liquid air and liquid oxygen to achieve the same effect. These three liquid gases were mostly employed to treat skin conditions like lesions, warts and keratosis (10). In 1950, Allington replaced the above gases with liquid N_2_ for the treatment of various skin diseases (11).

Rowbotham et al., developed a cannular device (a thin tube that can be inserted into a tissue), and employed it to perform cryoablation in patients with malignant brain tumors, by delivering a mixture of CO_2_ and acetone to the targeted region of the tissue (12). The major disadvantage with this technique is the non-insulation of the cannula, and therefore, passing a freezing agent through the inserted cannula not only caused a temperature drop in the targeted tumor, but also along the inserted tissue path. Later, Irving Cooper and Arnold Lee published landmark studies in the early 1960s that laid the foundation for current cryoablation technologies. They developed an insulated cannular device that could produce freezing effect only at the opening tip of the device, but not along its entire length, and successfully delivered −196°C liquid N_2_ to the localized tissue areas for treating patients (13). Later, many others used this technology to treat benign and malignant tumors successfully in patients. Gage et al., for example, recruited patients with benign and malignant tumors of the oral cavity, that were not suitable to receive the conventional modes of treatment like surgery and radiotherapy due to the reasons of tumor location or radio-resistance. Intriguingly, none of the patients treated with cryoablation showed local tumor recurrence. Modern cryoablation devices have replaced liquid N_2_ by an inert argon gas that produces the same effect (14–16), due to its associated technical advantages of easier handling and operation (9).

This review article summarizes the key cellular events and factors influencing the effectiveness of cryoablation, and discusses the potential combinatorial approaches of cryoablation with different forms of immunotherapy.

## Cardinals of Cryoablation

**Figure 1:**
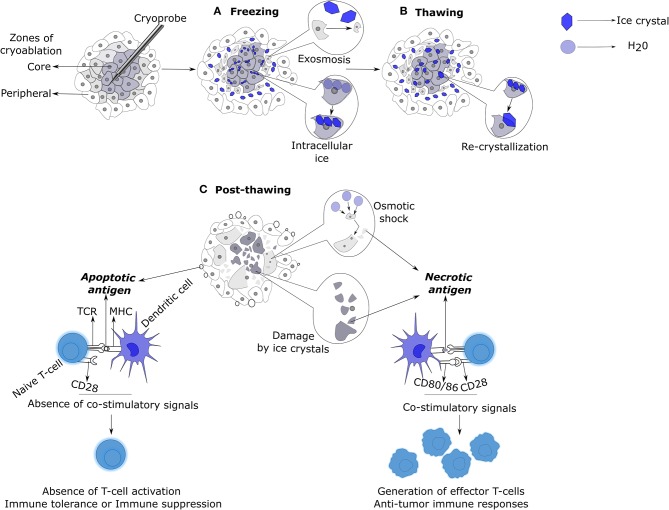
Mechanisms of cell death and immunologic responses induced by cryoablation. **(A)** Cells in the core of the ablation zone are subjected to lethal temperatures at rapid freezing rates, resulting in the generation of extra and intracellular ice crystals. Cells adjacent to the core zone undergo moderate or low freezing rates. This permits the cells to lose intracellular water by exosmosis in response to the formation of extracellular ice crystals resulting in cellular dehydration and shrinkage. In contrast, cells in the core zone cannot undergo exosmosis due to rapid freezing rates and thus, form intracellular crystals. Both intra and extracellular ice crystals cause mechanical damage to the cells. **(B)** During the thawing phase, the small intracellular ice crystals, due to their thermodynamic instability, fuse to form larger intracellular crystals (re-crystallization) that enhances the mechanical damage to the cell membranes and intracellular organelles. **(C)** Post-thawing, mechanically damaged cells die by necrosis and release their contents into the surrounding milieu. Cells that have undergone exosmosis swell and burst due to osmotic shock. Cells in the utmost periphery of the ablation zone exposed to sub-lethal temperatures undergo apoptosis, releasing apoptotic bodies. Antigens released from necrotic cells upon uptake by antigen presenting cells like DCs, induce co-stimulatory signals that would result in the generation of anti-tumoral T-cell responses. In contrast, antigen uptake by DCs in the form of apoptotic bodies imprints immune tolerance or anergy on T-cells due to the non-induction of co-stimulatory signals on DCs. DC, Dendritic cell.

Freezing rate is one of the most important factors that determines the kinetics and scope of tissue damage. At low freezing rates, solvents in extracellular spaces form ice crystals, leading to intracellular fluid loss as a compensatory mechanism for the osmotic imbalance across the cell membrane (17). This eventually leads to cell shrinkage, followed by damage to cell membranes and organelles due to the increased intracellular solute concentrations (Figure 1) (18). At high freezing rates, intracellular ice formation ensues the formation of extracellular crystals, because the cell does not have enough time to lose the solvent like above (17). This results in severe physical damage to cell membranes and intracellular organelles leading to cell death (18). Therefore, the faster the freezing the higher the level of intracellular ice formation, and the greater is the causation of cryolesion and tissue damage (7, 15, 17, 19, 20). The cells in close proximity to the cryoprobe undergo rapid freezing rates, whereas the cells in the periphery of the ablation zone are likely to undergo moderate or low freezing rates (7).

Different cell types undergo freezing at different temperatures, due to the variations in their intracellular solute compositions. For example, temperatures ranging from −4 to −7°C is enough to destroy the melanocytes by freezing, which can be resisted by the keratinocytes of the skin (21). In reference to the abundant early literature published on freezing temperatures tested on different healthy and tumor cell types, −50°C is the recommended temperature of freezing to ensure a definite tissue destruction (7, 16, 22).

The ideal duration of freezing is not defined, as the requirements vary based on the tissue type, vascularization, and the volume of the tissue to be frozen (7, 23). Duration of freeze should be evaluated in a manner that ensures the maximal freezing of the tumor tissue while sparring the surrounding normal tissue.

Upon thawing, the extracellular ice melts and causes local hypotonicity in relation to the dehydrated hypertonic intracellular environment. This leads to a high influx of solvent into the intracellular space, causing the previously dehydrated cells to swell and burst, releasing their intracellular contents into the surrounding milieu. At rapid cooling rates, the ice crystals formed are small and thermodynamically unstable; upon thawing, these small ice crystals fuse to form large crystals, a process known as re-crystallization (Figure 1). This process is maximized at −20 to −25°C during thawing. Large intra- and extracellular crystals thus formed cause severe mechanical damage to the lipid cell membranes. The slower the thawing, the higher the time for re-crystallization, and thus, greater the cellular damage by necrosis (7, 17).

The volume of tissue necrosis is directly proportional to the number of freeze-thaw cycles (22). The tissue that is already damaged in the first cycle of freeze-thaw goes through a faster cooling rate in the second cycle. In addition, there is also an increased formation of intracellular ice crystals in the additional cycle, all contributing to the severity of cell damage. It is estimated that about 80% of the previously ablated tissue is present in the succeeding cycle of ablation. Most studies employ two or more freeze-thaw cycles (7, 22, 24, 25). However, an increase in the number of freeze-thaw cycles and the proportional increase in tissue damage does not always translate into improved survival (26).

## Immunology of Cryoablation

The cells in the core of ablation zone that die by osmotic shock or physical damage (by ice crystals) die by necrosis, releasing their intracellular contents into the extracellular space, triggering an active immune response. In contrast, cells in the periphery of ablation zone subjected to sub-lethal temperatures die by a mitochondrial mediated apoptosis pathway (27, 28). The balance between immunogenic necrosis and immune tolerant apoptosis is one of the key factors that decides the course of an immune response induced by cryoablation (Figure 1). Freeze-thaw process also causes damage to the endothelial cells of the vasculature, leading to platelet activation, aggregation, thrombosis, and ischemia. This damage also causes edema, vasodilation, and hyperemia (7, 18, 27, 28).

There were reports in the 1970s of isolated cases having spontaneous regression of metastases upon cryoablation of primary prostate tumors. Results suggested that the regression could be immune mediated (29–32). Experiments with preclinical models bearing tumors treated by cryoablation exhibited an impressive resistance to tumor re-challenge (33–35). Comparison of cryoablation and surgery in a mammary tumor mouse model showed that 84% of the cryoablated mice resisted tumor re-challenge as opposed to 14% of mice treated by surgery. This enhanced protection was tumor-specific, as the survived mice were completely susceptible to re-challenge with an unrelated tumor cell line. Tumor rejection was immune mediated, as T-cells from the tumor draining lymph node (TDLN) of cryoablated mice secreted high levels of interferon-γ (IFN-γ) (36). Adoptive transfer of T-cells from the TDLNs of the cryoablation treated mice reduced lung metastases in the recipient tumor bearing mice; ~3-fold lower metastases was found in the recipients of T-cells from the cryoablation group as compared to the surgery group (37). These results provided a substantial evidence of immune-mediated protective responses evoked by cryoablation of tumors.

Den Brok et al. tested cryoablation on B16-OVA tumor-bearing mice. 50% mice were protected against tumor re-challenge when the cryoablated tumor was left *in situ*, whereas 100% death was observed when the cryoablated tumor was resected from the mice. This suggests that the ablated tumor tissue needs to remain *in situ*, where the tumor antigens will be available for the generation of an anti-tumor immune response (38). A study report of patients that underwent cryoablation for hepatic tumors exhibited increased levels of serum IL-6, serum amyloid A and C-reactive protein. A fraction of these patients also exhibited necrosis of distant untreated tumors, and were distinguished by increased levels of systemic IFN-γ and TNF-α (a Th1 response) in comparison to the patients that had only local necrosis of the cryoablated region. Interestingly, the latter group had increased levels of systemic IL-10, indicative of a Th2 response (39).

Taken together, above data point out to the fact that tumor cryoablation can trigger a tumor-specific protective immune response. However, the magnitude and sustainability of this immune response may not be adequate to protect from tumor re-challenge or exert an abscopal regression of distant metastases. In addition, tumors have developed sophisticated immune suppressive mechanisms to evade the host's immune attack (40–42). Therefore, synergy of cryoablation and immunotherapy to modulate and revert immunosuppressive responses presents a fascinating opportunity to treat cancer, in particular, for advanced metastatic cancer patients.

## Cryoablation Combined With Immunotherapy: Avenues for Immunomodulation

### Toll Like Receptors (TLRs)

TLRs are primarily expressed on innate immune cells like neutrophils, macrophages and dendritic cells (DCs), although expression on other cells has also been detected. These receptors recognize microbial-associated molecular patterns derived from microbiota and pathogens, and danger-associated molecular patterns derived from damaged and necrotic cells (43, 44). Each TLR has its own specific set of ligands; cell surface TLRs recognize lipids, proteins and lipoproteins, whereas intracellular TLRs recognize nucleic acid material. TLRs upon binding to their cognate ligands initiate a cascade of intracellular MyD88- or TRIF-mediated signaling pathways. The resultant of these signaling pathways is the production of type-1 interferons or other inflammatory mediators, initiating an immune response (44).

TLR agonist treatment with cryoablation in experimental tumor models has shown substantial improvements in survival and anti-tumor immune responses. Mice bearing less immunogenic B16-F10 tumors treated with cryoablation failed to survive tumor re-challenge. Fascinatingly, 50% of the mice survived upon re-challenge when the mice were prior treated with TLR-9 agonist CpG along with cryoablation. Analysis of tumor draining lymph nodes (TDLNs) demonstrated the DC maturation with an increased expression of CD80 and CD86 co-stimulatory markers (38). Another pre-clinical study employed imiquimod, a TLR7 agonist, along with cryoablation to treat B16-OVA tumors. Upon re-challenge, none of the mice survived when treated alone with imiquimod, and only 30–35% of the mice survived when treated with cryoablation. Intriguingly, 90% of the mice survived upon combination treatment with cryoablation and imiquimod (45). Patients with highly relapsing basal cell carcinoma treated with a combination of imiquimod and cryoablation demonstrated a 100% clinical response, with a 5% relapse rate observed at 18 months post-therapy, indicating a synergistic effect of the combined treatment modalities (46). Thus, TLR agonists can be employed as powerful adjuvants along with cryoablation in order to generate an effective antitumor immunity. Pro and anti-tumorigenic effects of each TLR on the specific cancer type targeted should also be taken into consideration for the selection of TLR agonists (43).

### Adoptive Cell Transfer Strategies

#### Dendritic Cells (DCs)

DCs are scattered across the body and act as a bridge between innate and adaptive immune responses. DCs in the tissues upon sensing TLR agonists undergo maturation, characterized by an increased expression of co-stimulatory molecules, migrate to the local draining lymph nodes for antigen presentation and induction of effector T-cell responses. In the absence of an immunogenic stimulus or a “danger signal,” DCs remain immature, and in such cases, antigen-presentation by DCs to T-cells can only cause T-cell tolerance (47). In the case of tumors, DCs upon TLR activation can mature, migrate from the tumor vicinity to TDLNs, and present the tumor antigenic peptides to T-cells, initiating an anti-tumor T-cell response (48). Moreover, DCs are efficient in inducing the differentiation of CD8^+^ T-cells to cytotoxic T-cells (CTLs) by a well-described mechanism known as cross-presentation (49). Generation of tumor-specific CTLs is the goal of most cancer immunotherapies (48).

Due to the low numbers of DCs available in the peripheral blood, they are generated *in vitro* from monocytes or bone marrow-mobilized hematopoietic precursors induced by granulocyte colony-stimulating factor administration (50). The majority of the adoptive transfer treatments have utilized autologous cells for the generation of DCs (51). Although the objective clinical response rates have been low ranging from 7 to 15%, an average of 20% increase in the overall survival has been observed (52).

The next-generation treatments aim to amalgamate DC-based therapies with other treatment modalities to achieve greater therapeutic responses (50, 51). In pre-clinical models of melanoma and lung carcinoma, tumor cryoablation followed by intra-tumoral DC transfer exhibited excellent improvements in the overall survival and resistance to re-challenge (53). In another study, a mouse model of colon cancer exhibited regression of distant untreated tumors upon combinatorial treatment of cryoablation and intra-tumoral administration of Bacillus Calmette-Guerin (BCG)-stimulated DCs. The systemic anti-tumor immunity that conferred protection was tumor-specific, and was abrogated by the depletion of CD8^+^ T-cells (54).

#### Natural Killer (NK) Cells

As the name indicates, these innate immune cells are specialized at killing virally infected or malignant cells. NK cells express activation and inhibitory receptors. Healthy host cells deliver equanimous activating and inhibitory signals to the NK cells. As a result, the healthy host cells are spared from killing. Cancer cells often lose the cell surface ligands that transmit inhibitory signals, and thus, NK cells receive only activating signals, leading to malignant cell killing. A surrogate mechanism of cytotoxicity occurs when a virally infected or a damaged cell expresses increased cell surface ligands that bind to NK cell activating receptors. NK cells can also recognize antibody-coated target cells owing to their expression of CD16/FcγRIIIa that binds to the Fc region of the IgG1 antibody, leading to antibody-dependent cell-mediated cytotoxicity (ADCC). NK cells lyse their targets by secreting cytolytic enzymes like perforin and granzyme. They also induce apoptosis via FAS and TNF- related apoptosis inducing ligand signaling induction in tumor cells. They secrete inflammatory cytokines like IFN-γ, TNF-α and IL-6 that facilitate the development of anti-tumor immune responses (55, 56).

A landmark study suggested that patients with decreased NK cell function exhibit increased incidence of cancers, indicating the role of NK cell function in controlling cancer development (57). *In vitro* expansion and transfusion of autologous NK cells in cancer patients was shown to be well-tolerated, but proved to be of no major clinical benefit (58). Later studies focused on treating cancers with adoptively transferred allogenic NK cells; haploidentical NK cells upon transfer did not give rise to graft-vs.-host disease complications in the recipients. This approach has resulted in a modest clinical success (59).

Some of the recent clinical studies tested the combination of cryoablation and allogenic NK cell transfer in patients with solid cancers. Although the data is preliminary, it demonstrates the synergistic effect of allogenic NK-cell infusions and cryoablation as compared to only cryoablation, in terms of clinical response rates. This beneficial effect has been shown in the patients with non-small cell lung cancer (NSCLC), hepatocellular and renal cell carcinomas, indicating the potentiating effect of NK cell therapy on cryoablation (60–62).

#### Cytokine Induced Killer (CIK) Cells

CIK cells are a heterogeneous population of cells obtained from *in vitro* cultures of cord blood or peripheral blood mononuclear cells with IFN-γ, anti-CD3 antibody and IL-2. The CIK population consists a majority of CD3^+^ CD8^+^ CD56^+^ NK-T cells that can recognize and kill cancer cells in a major histocompatibility complex (MHC)-independent manner, similar to NK cells. (63).

Repeated infusions of autologous CIK cells improved progression-free and overall survival as compared to cytokine treatments in patients with metastatic renal carcinoma (64). Triple-negative breast cancer patients that received CIK therapy in addition to chemotherapy exhibited improved rates of disease free and overall survivals (65). Similar results were also observed in a phase III clinical trial of patients with hepatocellular carcinoma (66).

Adoptive transfer of co-cultured DC-CIK cells were tested in combination with cryoablation in patients with metastatic hepatocellular carcinoma. Patients who received cryo-immunotherapy exhibited improved survival outcomes in comparison to patients who received only cryoablation or DC-CIK immunotherapy (67). Similar results were obtained in patients with metastatic pancreatic cancer (68). A triple combination regimen of cryoablation, chemotherapy and DC-CIK immunotherapy in metastatic NSCLC patients significantly improved survival outcomes as compared to patients who received other treatment regimens (cryo-immunotherapy, chemo-immunotherapy, cryo-chemotherapy, and only chemotherapy) (69). These clinical studies provide us with a definitive evidence of the enhancement in the therapeutic outcome by the inclusion of CIK immunotherapy along with cryoablation.

#### γδ-T Cells

These are a unique subset of T-cells with T-cell receptors (TCRs) composed of γ and δ chains, and recognize their ligands in a HLA/MHC-independent manner. They also express NK cell receptors like FcγR-III capable of mediating ADCC, and NKG2D capable of recognizing MHC-class I-related proteins and UL-16 binding proteins (70).

The Vγ9Vδ2-T cell population is the predominant subset of the γδ-T cell repertoire found in human peripheral blood, and commonly employed for γδ-T adoptive transfer treatments in clinical trials (71, 72). These cells directly recognize phosphorylated non-peptidic metabolites that are the byproducts of sterol and isoprenoid biosynthetic pathways, without the need for any intracellular processing and HLA-presentation. The increased phosphorylated antigen (pAgs) production by transformed or malignant cells is sufficient to activate Vγ9Vδ2-T cells, which does not occur in physiological conditions (71, 73). These cells can be extracted from patients and multiplied *in vitro* by utilizing synthetic pAgs (74) or anti-γδ TCR antibodies (75). Upon activation *in vivo* either by pAgs or by NKG2D-binding stress ligands, γδ-T cells respond rapidly akin to innate immune cells. Apart from exhibiting cytotoxic abilities similar to NK cells, they also can activate NK cells. The most intriguing of all, they acquire professional antigen presentation capabilities upon activation (72, 76, 77).

Vγ9Vδ2-T cells isolated from patients and expanded *ex vivo* exhibit excellent anti-tumor properties *in vitro*. Adoptive transfer into patients with renal cell carcinoma (78) or NSCLC (79) or other solid tumors (80) show safety and tolerability profiles, but no objective clinical responses. The disparity between *in vitro* and *in vivo* activities could be due to immuno-suppressive tumor microenvironment or a polarization from an anti-tumorigenic to a pro-tumorigenic cell type upon *in vivo* transfer (70, 81). It is worthwhile exploring the mechanisms of immunosuppression impinged on adoptively transferred Vγ9Vδ2-T cells in patients, and the possibilities to revert those mechanisms, as these cells already show a promise in pre-clinical settings (82, 83).

Often, tumors down regulate their MHC expression as an adaptation from evading immune recognition (84, 85). γδ-T cells due to their unique abilities, appear as ideal candidates for combating tumors in combination with therapies like cryoablation. It is encouraging to note that a recent study demonstrated that increased intra-tumoral infiltration of γδ-T-cells correlates with a better prognosis in a variety of human cancers (86). Cryoablation by inducing inflammation would create a suitable milieu for the adoptively transferred γδ-T cells to function effectively in the tumor bearing patients.

Currently, many clinical studies are underway to test the potential of various adoptive cell transfers along with cryoablative regimens as listed in Table 1, and improvement in the treatment outcomes are highly anticipated.

**Table 1:** Cryoablation combined with adoptive cell immunotherapies.

**NCT ID**	**Title**	**Type of immune therapy combined with cryoablation**	**Phase**
NCT02423928	Phase I clinical trial of cryoimmunotherapy against prostate Cancer (CryoIT)	DC adoptive transfer + Ipilimumab	Phase I
NCT03325101	Dendritic cell therapy after cryosurgery in combination with pembrolizumab in treating patients with stage iii-iv melanoma that cannot be removed by surgery	DC adoptive transfer + Pembrolizumab	Phase I/II
NCT03035331	Dendritic cell therapy, cryosurgery, and pembrolizumab in treating patients with non-hodgkin lymphoma	DC adoptive transfer + Pembrolizumab	Phase I/II
NCT02849366	Combination of cryosurgery and NK immunotherapy for recurrent sarcoma	NK cell adoptive transfer	Phase I/II
NCT02849327	Combination of cryosurgery and NK immunotherapy for recurrent pharyngeal cancer	NK cell adoptive transfer	Phase I/II
NCT02843802	Combination of cryosurgery and NK immunotherapy for advanced liver cancer	NK cell adoptive transfer	Phase I/II
NCT02843581	Combination of cryosurgery and NK immunotherapy for advanced esophageal cancer	NK cell adoptive transfer	Phase I/II
NCT02843815	Combination of cryosurgery and NK immunotherapy for advanced non-small cell lung cancer	NK cell adoptive transfer	Phase I/II
NCT02844335	Combination of cryosurgery and NK immunotherapy for advanced breast cancer	NK cell adoptive transfer	Phase I/II
NCT02849353	Combination of cryosurgery and NK immunotherapy for recurrent ovarian cancer	NK cell adoptive transfer	Phase I/II
NCT02849314	Combination of cryosurgery and NK immunotherapy for recurrent laryngeal cancer	NK cell adoptive transfer	Phase I/II
NCT02849379	Combination of cryosurgery and NK immunotherapy for recurrent tongue cancer	NK cell adoptive transfer	Phase I/II
NCT02849340	Combination of cryosurgery and NK immunotherapy for recurrent cervical cancer	NK cell adoptive transfer	Phase I/II
NCT02843607	Combination of cryosurgery and NK immunotherapy for advanced kidney cancer	NK cell adoptive transfer	Phase I/II
NCT03501056	Study of activated cytokine-induced killer armed with bispecific antibody for advanced lung cancer	CIK cell adoptive transfer + CD3-MUC-1 bispecific antibody	Phase II
NCT03484962	Study of activated cytokine-induced killer armed with bispecific antibody for advanced liver cancer	CIK cell adoptive transfer + CD3-MUC-1 bispecific antibody	Phase II
NCT03509298	Study of activated cytokine induced killer armed with bispecific antibody for advanced pancreatic cancer	CIK cell adoptive transfer + CD3-MUC-1 bispecific antibody	Phase II
NCT03540199	Study of activated cytokine-induced killer armed with bispecific antibody for advanced kidney cancer	CIK cell adoptive transfer + CD3-MUC-1 bispecific antibody	Phase II
NCT03524274	Study of activated cytokine-induced killer armed with bispecific antibody for advanced colorectal cancer	CIK cell adoptive transfer + CD3-MUC-1 bispecific antibody	Phase II
NCT03554395	Study of activated cytokine-induced killer armed with bispecific antibody for advanced gastric cancer	CIK cell adoptive transfer + CD3-MUC-1 bispecific antibody	Phase II
NCT03183219	Safety and efficiency of γδ T cell against liver cancer	γδ T cell adoptive transfer	Phase I/II
NCT03183232	Safety and efficiency of γδ T cell against lung cancer	γδ T cell adoptive transfer	Phase I/II
NCT03180437	Safety and efficiency of γδ T cell against pancreatic cancer	γδ T cell adoptive transfer	Phase I/II
NCT03183206	Safety and efficiency of γδ T cell against breast cancer	γδ T cell adoptive transfer	Phase I/II
NCT02380443	Increased frequency of allostim® immunotherapy dosing in combination with cryoablation in metastatic colorectal cancer	Adoptive transfer of activated allogenic Th1 memory cells expressing high levels of type 1 inflammatory cytokines, immunomodulatory molecules, and coated with CD3/CD28 microbeads	Phase II
NCT01741038	AlloStim® *in-situ* vaccine in pre-treated metastatic colorectal cancer	Adoptive transfer of activated allogenic Th1 memory cells expressing high levels of type 1 inflammatory cytokines, immunomodulatory molecules, and coated with CD3/CD28 microbeads	Phase II/III

### Immune Checkpoint Inhibitors

Following T-cell activation, an intracellular protein cytotoxic T-lymphocyte associated protein-4 (CTLA-4) is re-localized to the cell surface, and binds more avidly to CD80/86 molecules on APCs out competing CD28, effectively dampening the immune response. Thus, CTLA-4 plays the role of an immune checkpoint inhibitor, preventing a sustained immune response, and avoiding normal tissue injury. Genetic polymorphisms in CTLA-4 have been shown to be linked to a number of autoimmune diseases in humans, indicating its pivotal role in maintaining immune homeostasis (87). Another checkpoint inhibitor is programmed cell death-1 (PD-1), which is expressed on CD4^−^CD8^−^ thymocytes during T-cell development, CD4^+^ and CD8^+^ T-cells, B-cells and monocytes post-activation (87). PDL-1 and PDL-2 are the ligands of PD-1. PDL-2 exhibits a restricted expression on APCs, whereas PDL-1 is expressed on a variety of stromal and hematopoietic cell types. PDL-1 is also expressed by multiple myeloma, renal cell carcinoma, breast, ovarian, and pancreatic cancer types as well as many others. Engagement of PD-1^+^ T-cells with its ligands causes suppression of T-cell effector mechanisms, and induces T-cell exhaustion, thereby successfully counteracting the anti-tumor T-cell response (88, 89).

Tumor neo-antigen specific T-cells can be unleashed by antagonizing CTLA-4 and PD-1 checkpoints through blocking antibodies (90). Ipilimumab for CTLA-4, nivolumab and pembrolizumab for PD-1 are the commonly employed antibodies in pre-clinical and clinical settings (91).

Application of checkpoint blockers could be complemented with cryoablation in order to achieve durable therapeutic responses. In a murine prostate cancer model, all the mice bearing secondary tumors died despite receiving CTLA-4 blockade or cryoablation as a monotherapy; intriguingly, 44% of the mice survived when both therapies were given in combination (92). In a B16-OVA melanoma tumor model, CTLA-4 combined with cryoablation rescued 80% of the mice post tumor re-challenge as opposed to 40% only post cryoablation (93). These pre-clinical studies unambiguously exhibit a superior therapeutic outcome when cryoablation is applied along with checkpoint inhibitors. Preliminary data from pilot studies conducted in breast cancer and melanoma patients that received cryoablation and checkpoint inhibitors showed a good tolerability and a promise of efficacy (94, 95). Currently, various clinical trials are ongoing testing the potential of cryoablation and checkpoint blockade synergy in various cancers (Tables 1, 2).

**Table 2:** Cryoablation combined with immune checkpoint inhibitors.

**NCT ID**	**Title**	**Type of immune therapy combined with cryoablation**	**Phase**
NCT02833233	A study of pre-operative treatment with cryoablation and immune therapy in early stage breast cancer	Ipilimumab and nivolumab	Not applicable
NCT03546686	Peri-operative ipilimumab + nivolumab and cryoablation vs. standard care in women with triple-negative breast cancer	Ipilimumab and nivolumab	Phase II
NCT02489357	Pembrolizumab and cryosurgery in treating patients with newly diagnosed, oligo-metastatic prostate cancer	Pembrolizumab	Not applicable
NCT02626130	Pilot study of presurgical tremelimumab with or without cryoablation in patients with metastatic renal cell carcinoma	Tremelimumab	Phase I
NCT03189186	Phase-I Trial of pembrolizumab and percutaneous cryoablation combination followed by nephron-sparing surgery or cytoreductive nephrectomy in locally advanced and metastatic renal cell carcinomas	Pembrolizumab	Phase I
NCT02821754	A pilot study of combined immune checkpoint inhibition in combination with ablative therapies in subjects with hepatocellular carcinoma (hcc) or biliary tract carcinomas (btc)	Tremelimumab and durvalumab	Phase II
NCT03457948	Pembrolizumab and liver-directed therapy in treating patients with well-differentiated neuroendocrine tumors and symptomatic and/or progressive liver metastases	Pembrolizumab	Phase II

## Alliance of Cryoablation and Immunotherapy: Critical Aspects to be Addressed to Harness the Synergistic Therapeutic Potential

By cryoablating a local tumor, the immunogenic tumor antigens are released as a result of necrotic cell death, eliciting anti-tumor immune responses (96). However, cryoablation may trigger apoptosis, in particular at the periphery of the ablation zone where sub-lethal temperatures may be achieved, leading to anergy and clonal deletion. Factors influencing the balance between immunogenic necrosis and immunotolerant apoptosis induced upon cryoablation are ill-defined. Moreover, recent clinical studies report a fraction of patients that experience local tumor progression despite cryoablation, indicating the inadequacy of the local ablation in these patients (97, 98). Thus, it is critical to investigate the various aspects that are responsible to generate optimal local tumor control and anti-tumor immune responses by cryoablation. The aspects to be probed into include: the type of tumor (primary vs. metastasis) and the volume of tumor to be ablated, temperature, duration and number of freeze-thaw cycles, optimal number of ablations (one vs. multiple tumors and spacing between ablations), and to characterize the molecular pathways that are triggered by cryoablation in tumor and surrounding healthy tissues located within the treatment zone. It has been convincingly shown that despite the application of same ablation protocol on different organs, the zone of ablation differs from organ to organ due to their intrinsic attributes (99, 100). Hence, optimization of all the above listed parameters should be performed for each tissue or organ to be treated by cryoablation.

**Figure 2:**
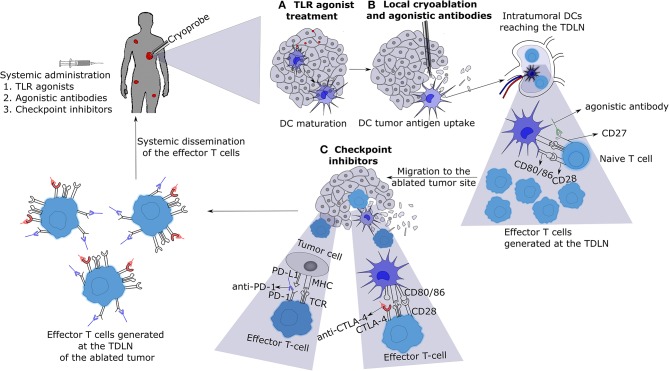
Cryo-immunotherapy: the patient with metastases is treated with local cryoablation of one of them combined with systemic immunotherapies. **(A)** Administration of low dose toll like receptor (TLR) agonists will cause the activation and maturation of DCs. **(B)** Cryoablation of a tumor induces tumor necrosis and release of tumor antigens into the surrounding milieu, which are taken up by mature DCs located near the tumor or TDLN. These DCs present tumor antigens to naive T-cells in the TDLN in the presence of agonistic antibodies (for example, CD27) leading to enhanced activation and differentiation into effector T-cells. **(C)** The effector T-cells thus generated will migrate to the cryoablated tumor site encountering the tumor cells and DCs presenting tumor antigens on their surface MHC molecules. Introduction of checkpoint inhibitors (for example, anti-CTLA-4 and anti-PD-1) will allow the T-cells to execute tumor cell killing without being inhibited by the checkpoint signaling. Eventually, the effector T-cells with blocked checkpoint molecules will also migrate to the distant metastasized tumor sites, leading to the regression of metastases. TLR, toll like receptor; DC, dendritic cell; TDLN, tumor draining lymph node; TCR, T-cell receptor; MHC, major histocompatibility complex.

Systemic immune responses induced by local tumor cryoablation, in most cases, is not of magnitude enough to cause regression of untreated distant tumors. However, the systemic immune responses generated by cryoablation can be exploited for the application of various immunotherapies (Figure 2). Checkpoint inhibitors such as anti-CTLA-4, anti-PD-L1, and anti-PD-1 combined with cryoablation appear particularly appealing. Other checkpoint inhibitors against molecules like Tim-3, Vista, Lag-3, TIGT, CD276, and BTLA should also be tested in combination with cryoablation (101). Moreover, immune agonistic antibodies directed at molecules such as CD27, CD40, OX40, 4-1BB, and ICOS should also be experimented (101, 102). Various immunotherapies must be screened in combination with cryoablation on different tumor pre-clinical models in order to perceive which labyrinth combinations will yield the best outcome for each tumor type. Some facets of crucial importance to be delved into are: (a) the optimal dosage of each immunotherapy, (b) the ideal route of administration, and (c) the right timing or scheduling of each treatment to achieve maximal anti-tumor effects, while minimizing treatment toxicities.

Despite the beneficial effects of systemically applied immunotherapies, many of them have their associated toxicities due to the systemic disruption of immune homeostasis. If a treatment consists of one or more immunotherapies administered systemically along with local cryoablation, it could lead to toxicity due to the cumulative toxic off-target effects of each therapy. An alternative method of therapy highly promising is to apply both cryoablation and immunotherapy in a local manner, with an aim of achieving a systemic abscopal effect. Local application will empower the clinicians to exercise various immunotherapies in combination, while evading toxicities. The idea here is that the locally initiated anti-tumor immune responses will disseminate systemically to regress the distant untreated tumors (103, 104). A key aspect to be evaluated is whether to restrict multiple rounds of local immunotherapy to a single tumor site or to deliver each round of therapy to a different tumor site. Although preliminary, encouraging data from patients demonstrated that by treating the primary tumors with cryoablation combined with intratumoral injections of anti-CTLA4 and anti-PD-1, distant metastasized tumors exhibited regression (105). Future cancer treatments might constitute an application of both local and systemic immunotherapies alongside cryoablation, in order to minimize the toxicities while enhancing the anti-tumor immune responses.

Tumors that have a low mutational burden and are non-immunogenic are often referred to as cold tumors (106). Local delivery of cryo-immunotherapy might not be sufficient to generate distant abscopal anti-tumor effects. In such cases, there is a need for an increased exploration of adoptive CIK cell, NK cell, and γδ-T cell immunotherapies in combination with cryoablation. These alternatives have been less explored in solid cancers as opposed to the adoptive transfers of chimeric antigen receptor (CAR) T-cells and tumor immune infiltrating T- cells (TILs). TILs have an obligation of MHC/HLA restriction, which limits their functional ability when the tumor cells downregulate MHC expression (85, 107). CARs, although being not MHC restricted, require a homogenous expression of the targeted tumor antigen on the entire tumor cell population, a prerequisite not met in most cases, unlike hematologic neoplasms (108). The anti-tumor functions of CIK cells, NK cells, and γδ-T cells are neither curtailed by MHC downregulation nor by the lack of homogenous antigen expression on tumors. Thus, these cell types present as attractive and contemporary alternatives to be considered into the treatment planning in future. Further research is required to identify the conditions that make these cell populations exert their anti-tumoral capacities at their optimum *in vivo*, and then coalesce with cryoablation modality to reap better objective clinical responses than the currently available treatments.

The regimen of cryoablation and the combinatorial choice of immunotherapies should be personalized with respect to the individual patient's constitution. The two key components to be considered are the tumor mutational burden with its corresponding immunogenicity and the patient's immune constitution. Immune signatures vary across patient populations. For example, due to a difference in the immunogenetic constitution, one patient treated with a cryo-immunotherapy protocol might generate an optimal systemic anti-tumor immune response, and another might generate a sub-optimal or in the worst cases, a pro-tumorigenic response. Therefore, to address the problem of inter-patient variability, blood and tissue samples should be analyzed in-depth for their overall immune constitution prior and at different time points post treatment, and for the corresponding changes, if any, in the systemic and locoregional immune make-up post treatment. This will provide a peek into the kind of immune infiltrate that congregates at the tumor site as a response to a specific therapy delivered. Besides, tumors are heterogeneous and exhibit different clonal evolution patterns within a patient during disease progression (109). Hence, differences may exist across tumors lesions (intra-patient variability) and a treatment, which works on a group of metastases, may prove to be innocuous for other lesions. Thus, tumor biopsies should be taken at every treated site (and in lesions evading treatment) at the time of cryoablation to account for biologic variability and adapt subsequent treatments accordingly.

In conclusion, the use of cryoablation as an *in vivo* vaccination tool has far-reaching implications beyond the treatment of local tumors. Owing to its ability to elicit anti-tumor immune responses and occasional abscopal regression of tumor metastases, cryoablation combined with targeted immunotherapies could evolve as the best modus operandi to treat the patients with advanced tumor metastases. Meticulous efforts to determine the optimal conditions for cryoablation in the context of generating an effective immune response, along with identifying its ideal immunotherapy consorts are the need of the hour. Eventually, refining the cryo-immuno regimen to a crux where it will be tailor made for each patient should be a part of our future endeavors to achieve improved treatment outcomes for cancer patients.

## Author Contributions

CY and RD wrote the first draft of the manuscript. CC, LK, and AD contributed to some sections of the manuscript. All authors contributed to manuscript revision and approved the submitted version.

### Conflict of Interest

The authors declare that the research was conducted in the absence of any commercial or financial relationships that could be construed as a potential conflict of interest.
